# Prebiotic xylo-oligosaccharides cause modest taxonomic shifts in the gut microbiota but do not increase insulin sensitivity in insulin resistant individuals

**DOI:** 10.1186/s12986-026-01168-3

**Published:** 2026-07-02

**Authors:** Anastasiia Driuchina, Jenni Hekkala, Jukka Hintikka, Perttu Permi, Ella Hellsten, Sanna Lensu, Viktor Bielik, Jari Laukkanen, Satu Pekkala

**Affiliations:** 1 Faculty of Sport and Health Sciences, University of Jyväskylä, PO Box 35, Jyväskylä, FI-40014 Finland; 2 Institute of Biomedicine, University of Eastern Finland, Kuopio, Finland; 3 Department of Biological and Environmental Science, Nanoscience Center, University of Jyväskylä, Jyväskylä, Finland; 4 Department of Chemistry, Nanoscience Center, University of Jyväskylä, Jyväskylä, Finland; 5 Institute of Biotechnology, Helsinki Institute of Life Science, University of Helsinki, Helsinki, Finland; 6 Centre for Interdisciplinary Brain Research, Department of Psychology, University of Jyväskylä, Jyväskylä, Finland; 7 Faculty of Physical Education and Sport, Department of Biological and Medical Sciences, Comenius University, Bratislava, Slovakia; 8 Institute of Clinical Medicine, University of Eastern Finland, Kuopio, Finland; 9 Wellbeing Services County of Central Finland, Jyväskylä, Finland

**Keywords:** Gut microbiota, Fecal metabolites, Prebiotic supplementation, Inflammation, Body composition, Insulin sensitivity, Lipid profile

## Abstract

**Background:**

Overweight increases the risk of Type 2 Diabetes (T2D), and insulin resistance (IR) precedes development of T2D. Besides lifestyle factors, gut microbes and their metabolites contribute to IR. Gut microbiota could be modulated by probiotics and prebiotics to improve IR, but current research results from interventions are controversial. We aimed at exploring the effects of prebiotic xylo-oligosaccharides (XOS) on the structure and metabolites of gut microbiota, IR, body composition and inflammatory response in humans with overweight.

**Methods:**

In this within-subjects study design the participants had a 1-month baseline control period without changing their lifestyle and then, they were subjected to 4-months of dietary XOS supplementation. Based on baseline HOMA-IR, we divided study participants into insulin sensitive (< 3, IS, *n* = 11) and insulin resistant (> 3, IR, *n* = 38) groups. The gut microbiota were analyzed using 16 S rRNA gene sequencing. Fecal metabolomes were quantified using nuclear magnetic resonance spectroscopy (NMR). Additionally, blood clinical variables, serum cytokines, body composition and diet were studied.

**Results:**

We identified clear differences between insulin-resistant (IR) and insulin-sensitive (IS) participants in gut microbiome, body composition, and glucose–insulin profiles, along with modest variations in proinflammatory cytokines. Although four months of XOS supplementation did not alter insulin sensitivity or most other measured outcomes, it slightly reduced the levels of total cholesterol and increased HDL cholesterol in IR participants. However, it should be noted that during control period HDL first decreased in that group. Interestingly, XOS treatment decreased fecal galactose, isobutyrate, and isovalerate levels in IS participants, whereas shifts in microbial abundances (20 different microbial taxa) were observed only in IR participants with no changes in diversity. Isobutyrate, isovalerate and phenylacetate levels were lower in the IR group at baseline compared to IS. Across the study from baseline to post-treatment, body weight and BMI declined in IR individuals.

**Conclusions:**

XOS supplementation altered gut microbiota composition in IR participants, while changing fecal metabolome in IS participants. XOS modestly improved lipid parameters, such as HDL cholesterol, but did not improve insulin sensitivity, body composition, or inflammatory markers. More and larger studies are needed to show that prebiotic XOS can consistently improve insulin sensitivity.

**Trial registration:**

The study was retrospectively registered at ISRCTN under the number ISRCTN86495943.

## Background

The obesity epidemic worsens constantly, and over 890 million adults were living with obesity (Body mass index, BMI > 30) in 2022 (WHO). The worldwide number of individuals with overweight (BMI > 25) is 2.5 billion adults, and they are around three times more likely to develop Type 2 Diabetes (T2D) than normal weight individuals, whereas a meta-analysis of 30 studies found that ~ 77% of individuals diagnosed with T2D were living with obesity [1].

Insulin resistance (IR) and decreased insulin sensitivity precede development of T2D though not all underlying mechanisms are known [2]. Clearly, lifestyle factors such as diets high in added sugars and physical inactivity predispose to IR and T2D [3, 4]. However, growing evidence shows that gut microbes and various metabolites that they produce influence the onset of IR [5]. Special attention is paid to gut bacteria producing metabolites such as short-chain fatty acids (SCFAs) and bile acids, which play a role in insulin signaling and contribute to IR [6, 7]. A cross-sectional analysis of two population-based prospective cohorts has shown that also higher microbiome diversity and richness were associated with less IR [8].

The gut microbiota community composition and functions can be modulated by probiotics and prebiotics that thus might be used to ameliorate a disease to some extent. Probiotic refers to live microorganisms, e.g. like certain bacteria or yeasts that, when consumed in adequate amounts, provide a specific health benefit to the host [9]. Prebiotics serve as a food for beneficial gut microbes conferring health benefit to the host [10]. Many interventions in humans have shown positive effects of probiotics on glucose control but also many have shown no effects or even adverse effects [11]. Interventions on chronic noncommunicable diseases such as T2D in humans show many inconsistent findings on the effects of prebiotics such as fructo-oligosaccharides and inulin on glycemic control [11]. However, some studies indicate that resistant starch can improve insulin sensitivity in healthy subjects [12] and in subjects with overweight and obesity [13]. A small trial with randomized double-blind clinical design in T2D patients showed that dietary supplementation with prebiotic xylo-oligosaccharides (XOS) was effective in improving the blood sugar and lipids [14], while another study failed to do so [15].

Due to the above-presented controversial findings on the effects of prebiotics on insulin sensitivity and on our own results [16] and of others [17] showing the growth of health beneficial bacteria by XOS supplementation, we aimed at exploring the effects of XOS on the structure and metabolites of gut microbiota, clinical biomarkers of T2D, body composition and inflammatory response in humans with overweight, paying special attention to IR.

## Methods

### Study participants and intervention

The study got a positive statement from the Ethics Committee of the Hospital District of Southwest Finland (currently Wellbeing Services County of Southwest Finland) (ETMK 72/2019). The participants were recruited by advertising in social media (Facebook, Instagram) and in the web pages of the University of Jyväskylä, Finland. The inclusion criteria were an age between 18 and 75 years, being overweight (BMI > 25 kg/m^2^), and high waist circumference (> 102 cm for males, > 88 cm for females). The exclusion criteria were antibiotic treatment one month prior to the study, inflammatory bowel disease, celiac disease, major eating disorders, and hypothyroidism. A written informed consent was collected from all participants prior to the study.

We recruited 49 participants (25 males/24 females) for the within-subjects designed intervention with a baseline control period; each participant underwent a one-month control phase before commencing the XOS prebiotic intervention. Due to the known interindividual variation in the gut microbiota, this is a better approach to see changes in the gut microbiota than using a separate control group [18]. During the intervention, the participants were given daily doses, 2.8 g of XOS powder in small sachets to be consumed daily dissolved in juice or yogurt for four months. XOS was purchased from Van Wankum Ingredients (Maarssen, Netherlands) and was 95% pure. While there is no clear consensus of the optimal amount of daily XOS intake, the current recommendation of 2.8 g/day is based on the original article by Finegold et al. 2014 [19]. Similarly, Na & Kim showed that 2.8 g/day of XOS caused a more pronounced prebiotic effect than 1.4 g/day [20]. Also, Kajihara et al. reported that 3 g/day of XOS promoted growth of Bifidobacteria, while decreasing blood ammonia levels in patients with liver cirrhosis [21].

Throughout both the control and intervention periods, the participants were instructed to maintain their usual diet and physical activity levels. 41 participants (22 males/19 females) completed the study. The few dropouts were mainly due to lack of time or other personal reasons.

The participants were divided into insulin sensitive (IS) and insulin resistant (IR) group based on their Homeostatic Model Assessment for Insulin Resistance (HOMA-IR) results. Blood samples were taken after overnight fasting (see below). The analysis of glucose was performed using Indiko clinical chemistry system (Thermo, Vantaa, Finland). Immulite 2000 immunochemistry system (Siemens Healthineers, Erlangen, Germany) was used for insulin analysis. HOMA-IR was calculated as fasting insulin (mU/L) x fasting glucose (mmol/L)/22.5. A subgroup of the participants with baseline HOMA-IR < 3 was classified as IS (*n* = 11), while participants with baseline HOMA-IR > 3 were classified as IR (*n* = 38). These values were based on a large Finnish population study [22] as there are no clear cutoff values for HOMA-IR in clinical use or in the scientific literature.

### Body composition measurements, oral glucose tolerance test, clinical variables and analyses of serum cytokines

Body composition was assessed using dual x-ray absorptiometry (DXA, Lunar Prodigy, GE Healthcare, Madison, WI, USA) and bioimpedance (InBody, Seoul, South Korea). Weight was measured with bioimpedance. Height was measured using a wall-fixed measuring device. BMI was calculated as weight (kg)/height^2^ (m). Waist circumference was measured midway between the lowest rib and the iliac crest twice with a tape measure and the mean value was used for the analyses.

Blood samples were collected at each time point after an overnight fast by drawing into 10 mL coagulation activator tubes (Vacutest 11030). Serum was separated by centrifugation at 2000 x g for 10 min and stored in aliquots at -80° C until analysis. The analysis of serum clinical variables was performed using Indiko clinical chemistry system (Thermo, Vantaa, Finland). Specifically, liver enzymes (AST, ALT, GGT), total cholesterol, HDL, LDL, free fatty acids, and triglycerides were measured.

Oral glucose tolerance test (OGTT) was done at baseline and after the XOS intervention. For OGTT, a basal fasting blood sample was taken to measure blood glucose levels. Then, the participants drunk 75 g of glucose solution. Blood samples were taken at 30, 60 and 120 min after consuming the glucose solution to measure blood glucose levels using Indiko clinical chemistry system. The serum cytokines were analyzed using a high sensitivity human 15-plex-plex Quansys ELISA assay (#112433HU, Quansys Biosciences, Logan, UT, USA). The assay was conducted and then quantified with Quansys instrument and Q-View software (Quansys Biosciences) as specified in the manufacturer’s instructions. The detection limits were as follows: Interleukin (IL)-1α 0.8 pg/ml, IL-1β 2.3 pg/ml, IL-2 0.032 pg/ml, IL-4 0.012 pg/ml, IL-5 0.18 pg/ml, IL-6 0.16.pg/ml, IL-10 1.59 pg/ml, IL-12 1.7 pg/ml, IL-13 0.2 pg/ml, IL-15 2.3 pg/ml, IL-17 3.09 pg/ml, IL-23 28.29 pg/ml, Interferon (IFN)γ 0.064 pg/ml, Tumor necrosis factor (TNF)α 0.44 pg/ml, 0.27 TNFβ pg/ml.

### Diet analyses

The diet was analyzed from 3-day self-reported food diaries (2 weekdays and 1 weekend day) using Fineli database and food diary analysis web tool (https://fineli.fi). An average daily intake of energy and nutrients (except the intake of XOS, which was 2.8 g per day) were calculated from the 3 days. Fecal sampling and food diary filling were performed within the same week. Food diaries were collected at each timepoint.

### Collection and analysis of the fecal samples

The participants collected the fecal samples at their homes in regular plastic collection tubes, which were frozen at domestic freezers immediately after collection, brought to the laboratory frozen within two days after the sampling. Samples were stored immediately at − 80°C. Total DNA extraction from the fecal samples was performed as described previously [23]. Briefly, the DNA was extracted from ~ 80 mg of feces with Stool Extraction Kit and semi-automated GenoXtract instrument (Hain Lifescience GmbH, Nehren, Germany), combined with preceding homogenization with bead-beating in 0.5/1.0 mm Ceramic Bead Tubes (Zymo Research, Irvine, CA, USA). Then, for microbial community analysis, 16S rRNA gene was amplified using primers S-D-Bact-0341-b-S-17, 5´-CCTACGGGNGGCWGCAG-3’ and S-D-Bact-0785-a-A-21, 5´-GACTACHVGGGTATCTAATCC-3´ targeting the V3-V4 regions of the SSU rRNA gene. In the first PCR, the reaction consisted of 1xMaxima™ SYBR Green qPCR Master Mix (Thermo Fisher Scientific, Waltham, MA, USA), 10 µM of primers and 20–80 ng of DNA template. Thermal cycling consisted of 10 min initial denaturation at 95 °C, 30 cycles at 94 °C 30 s, + 52 °C 60 s and 72 °C 60 s and final extension at 72 °C for 5 min (C1000 ThermalCycler, Bio-Rad Laboratories, Hercules, CA, USA). To add Ion Torrent PGM sequencing adapters and barcodes to the ends of the PCR product, one µl of the PCR product was used as template in the second PCR, where 10 cycles were performed using linker and fusion primers (10 µM of M13_ S-D-Bact-0341-b-S-17, 1 µM of IonA_IonXpressBarcode_M13 and P1_ S-D-Bact-0785-a-A-21), with conditions otherwise identical to the first amplification. PCR products were purified with AMPure XP (Beckman Coulter, Brea, CA, USA), quantified with PicoGreen (Quant-iT™ PicoGreen™ dsDNA Assay Kit, Thermo Fisher Scientific), and pooled in equimolar quantities for sequencing on Ion Torrent PGM using Hi-Q View OT2 Kit for emulsion PCR, Hi-Q View Sequencing Kit for the sequencing reaction, and Ion 318 Chip v2 (Thermo Fisher Scientific). After that, the 16 S rRNA gene sequences were quality-filtered and clustered to operational taxonomic units (OTUs) at the 97% similarity using CLC Microbial Genomics Package (Qiagen, Hilden, Germany). The 16 S rRNA gene sequences were classified using SILVA SSU Ref database (v138.1, 99%).

### NMR spectroscopy for quantification of fecal metabolites

NMR spectroscopy was used to characterize small metabolites in fecal suspensions. Cold phosphate-buffered saline (pH 7.4) was mixed with the feces at a ratio of 1:2 and vortexed for 5 to 10 min to extract the metabolites. The extract was then centrifuged at maximum speed for 15 min at 4 °C. A part of the resultant supernatant was mixed with Chenomx standard solution (5 mM deuterated DSS (DSS-d6) and sodium azide in D2O) in 1:10 ratio and vortexed. Then, the prepared mix was placed into 3 mm O.D. round bottom NMR tubes. NMR data were collected using a Bruker Avance HD III NMR spectrometer, operating at 800 MHz of 1H frequency, and equipped with a cryogenically cooled ^1^H, ^13^C, ^15^N TCI probehead. A so-called 1D NOESY experiment with residual water signal presaturation was employed to obtain quantitative 1H spectra. Spectra were collected with 32 transient per free induction decay (FID), using the recycle delay of 5 s, with 4 s acquisition time. Isotropic mixing time was set to 10 ms and the excitation sculpting with gradients was employed to the water signal suppression [24]. The ^1^H NMR spectra were phase-, baseline- and shim-corrected and referenced to the internal standard at 0.00 ppm using Chenomx NMR Suite (Chenomx Inc., Edmonton, Alberta, Canada). The metabolites were identified and quantified using Chenomx with the aid of databases derived from Human Metabolome Database (HMDB, http://www.hmdb.ca), and Biological Magnetic Resonance Data Bank (BMRB, http://www.bmrb.wisc.edu).

### Statistical analysis

Statistical analyses of body composition, clinical variables, OGTT results, cytokines, fecal metabolites and diet were performed using IBM SPSS Statistics version 28 (Armonk, New York, USA). The number of fecal metabolites quantified was 49, and multiple test correction was not applied due to low power and collinearity in the fecal sample dataset. Normal distribution of the variables was analyzed using Shapiro Wilk’s test, skewness and kurtosis. At each timepoint, group differences in body composition, clinical variables, dietary intakes and fecal metabolites were analyzed with MANOVA and estimated marginal means, except that after the control period and after XOS the group differences in body composition were analyzed with ANCOVA using the corresponding baseline value as covariate and Bonferroni as post hoc test. Longitudinal changes in body composition, clinical variables and dietary intakes were analyzed using General Lineal Model repeated measures with Bonferroni post hoc correction. Longitudinal changes in fecal metabolites and serum cytokines were assessed using Wilcoxon signed-rank test, and group differences in cytokines at each timepoint using Mann Whitney U test. The statistical significance was set at *p* < 0.05.

Statistical analysis of the gut microbiota was conducted with CLC Microbial Genomics package (Qiagen). Low abundance OTUs (combined abundance less than 10) were filtered out before analyses. To analyze gut microbiota alpha-diversity, Chao1 (species richness) and Shannon (species diversity) indices were quantified. In addition, phylogenetic diversity was determined. The differences in the alpha- and phylogenetic diversity measures between the groups and time points were analyzed with Kruskal-Wallis test. Beta-diversity (the dissimilarity between samples) analysis was based on Bray-Curtis dissimilarity and PERMANOVA (group, time and group-time interaction) for significance testing. The number of permutations was 99,999. The taxonomic differences between the groups and timepoints were analyzed with Generalized Linear Model (GLM) comparison in CLC Microbial Genomics Package (Qiagen), followed by correction for Benjamini-Hochberg false discovery rate (FDR). A full explanation of the statistical model can be found in the Qiagen manual [25]. In the GLM analyses, statistical significance was set at FDR < 0.05.

### Use of large language models (LLMs)

Microsoft Copilot was used to polish English language in some places of the text and to help in finding some references.

## Results

### Body composition and blood values

**Fig. 1:**
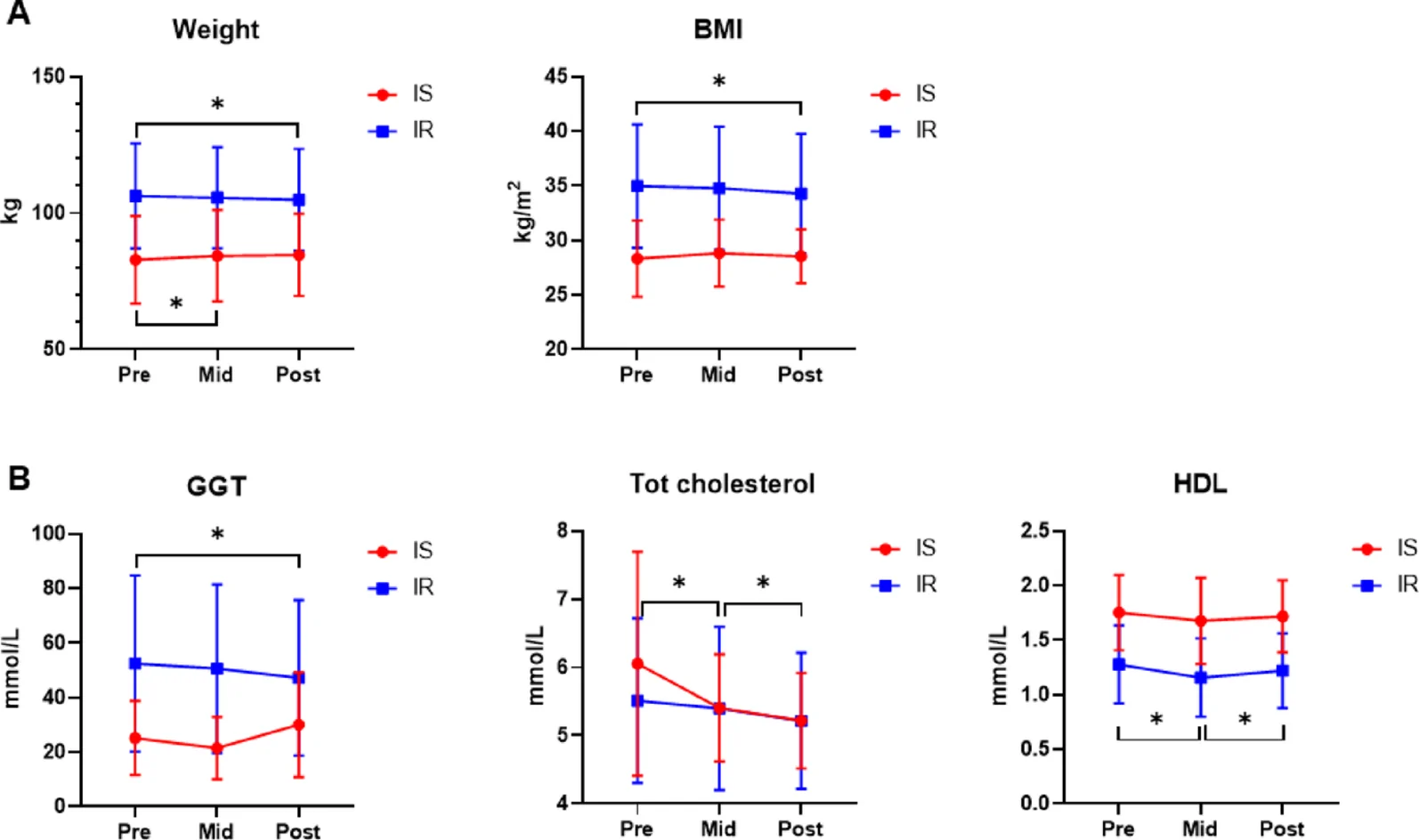
Significant changes in body composition and clinical variables within the groups across timepoints. **A**) Weight (kg) and BMI (kg/m^2^) changes across the timepoints. **B**) GGT, total cholesterol and HDL (all mmol/L) changes across the timepoints. Pre= baseline, Mid= after the control period/before XOS, Post= after XOS treatment. Longitudinal changes were analyzed using General Lineal Model repeated measures with Bonferroni *post hoc* correction. * *p* < 0.05

Out of 49 subjects enrolled in this study, 11 subjects were insulin sensitive (IS) and 38 were insulin resistant (IR). The mean (± SD) age was 53 (± 11) years in the IS group and 54 (± 8) years in the IR group with no statistical difference between the groups. No difference in sex distribution was found between the groups. Table 1 presents body composition and clinical parameters of the IS and IR groups across all time points. Significant differences were observed in all body composition variables between the IS and IR groups at baseline (*p* < 0.05 for all, Table 1). Therefore, to study group differences in body composition after the control period and post-treatment, the corresponding baseline variable was used as covariate in ANCOVA. After the control period with covariate adjustment, the groups did not differ from each other in any of the body composition variables (Table 1), but after the XOS treatment the IR group had higher weight (*p* = 0.013), BMI (*p* = 0.033), waist circumference (*p* = 0.044), visceral fat area (*p* = 0.003) and total fat mass (*p* = 0.015) than the IS group (Table 1). In the IS group, body composition variables remained stable across timepoints. However, in the IR group, there was a slight decrease in weight (*p* = 0.043) and BMI (*p* = 0.019) from the baseline to after the XOS treatment (Fig. 1A).

Regarding clinical parameters, insulin and HOMA-IR levels were significantly higher in the IR group at all timepoints, while HDL levels were lower (*p* < 0.05 for all, Table 1). Triglycerides were higher in IR group only at baseline (*p* = 0.012, Table 1). The Matsuda index indicating insulin sensitivity and calculated at baseline, was significantly lower in the IR group (*p* < 0.001, Table 1). Among liver enzymes, ALT levels were higher in the IR group at baseline, and GGT levels were higher both at baseline and after the control period (*p* < 0.05 for all, Table 1). No significant changes were observed across timepoints in the IS group, while in the IR group, GGT and total cholesterol levels slightly decreased from baseline to control period and further to post-intervention (*p* < 0.05 for all, Fig. 1B). HDL increased in the IR group during the XOS treatment (*p* = 0.003, Fig. 1B). However, it should be noted that HDL had previously decreased during the control period (*p* < 0.001, Fig. 1B).

**Table 1:** Body composition and clinical variables (mean ± SD) as well as the differences between the groups at different timepoints of the study

	PRE			MID			POST		
	**IS** (*n* = 11)	**IR** (*n* = 38)	*p*	**IS** (*n* = 8)	**IR** (*n* = 36)	*p*	**IS** (*n* = 7)	**IR** (*n* = 34)	*p*
**Body composition**									
Weight (kg)	82.9 ± 16.1	106.3 ± 19.2	**< 0.001**	84.3 ± 16.8	105.6 ± 18.5	0.300^#^	84.7 ± 15.0	104.9 ± 18.7	**0.013** ^**#**^
BMI	28.3 ± 3.5	34.9 ± 5.7	**< 0.001**	28.8 ± 3.1	34.8 ± 5.7	0.776^#^	28.5 ± 2.5	34.3 ± 5.5	**0.033** ^**#**^
WC (cm)	101.7 ± 9.1	118.7 ± 13.3	**< 0.001**	101.6 ± 7.4	117.3 ± 12.7	0.578^#^	100.9 ± 5.9	116.9 ± 12.5	**0.044** ^**#**^
Visceral fat (cm^2^)	131.8 ± 23.3	189.5 ± 42.5	**< 0.001**	139.5 ± 20.6	185.0 ± 37.3	0.761^#^	128.2 ± 22.3	181.0 ± 39.2	**0.003** ^**#**^
Total fat (kg)	28.7 ± 8.6	43.5 ± 12.1	**< 0.001**	31.5 ± 8.2	42.6 ± 11.9	0.768^#^	30.5 ± 6.1	40.2 ± 10.3	**0.015** ^**#**^
Android fat (kg)	2.88 ± 0.78	5.13 ± 1.75	**< 0.001**	3.03 ± 0.74	4.99 ± 1.65	0.260^#^	2.92 ± 0.63	4.64 ± 1.31	0.079^#^
Gynoid fat (kg)	4.89 ± 2.01	6.43 ± 2.00	**0.030**	5.52 ± 1.94	6.34 ± 2.08	0.310^#^	5.30 ± 1.53	5.96 ± 1.84	0.347^#^
**Blood**									
Tot chol (mmol/L)	6.05 ± 1.64	5.51 ± 1.21	0.235	5.41 ± 0.79	5.40 ± 1.20	0.990	5.22 ± 0.70	5.21 ± 1.00	0.993
LDL (mmol/L)	3.25 ± 0.70	3.31 ± 0.96	0.864	3.38 ± 0.49	3.37 ± 1.07	0.983	3.37 ± 0.54	3.35 ± 0.91	0.954
HDL (mmol/L)	1.75 ± 0.35	1.28 ± 0.36	**< 0.001**	1.68 ± 0.39	1.16 ± 0.36	**< 0.001**	1.72 ± 0.33	1.22 ± 0.34	**0.001**
Trigly (mmol/L)	1.00±0.037	2.12 ± 1.40	**0.012**	0.99 ± 0.36	2.33 ± 1.94	0.061	1.03 ± 0.48	2.20 ± 1.66	0.076
FFA (mmol/L)	583.1 ± 281.3	689.1 ± 362.9	0.378	623.7 ± 385.9	568.29 ± 260.0	0.621	560.8 ± 255.7	559.8 ± 144.9	0.988
Glucose (mmol/L)	5.83 ± 0.56	6.42 ± 0.93	0.053	6.37 ± 1.28	6.21 ± 0.54	0.554	6.08 ± 1.13	6.25 ± 0.52	0.535
Insulin (IU)	4.52 ± 2.15	24.25 ± 12.55	**< 0.001**	11.69 ± 8.87	22.41 ± 10.43	**0.007**	6.67 ± 4.64	22.14 ± 12.34	**0.003**
HOMA-IR	1.19 ± 0.58	7.01 ± 3.82	**< 0.001**	3.73 ± 3.73	6.20 ± 2.94	**0.040**	1.76 ± 1.22	6.17 ± 3.40	**0.002**
Matsuda index	8.31 ± 1.98	2.63 ± 1.35	**< 0.001**	-	-		-	-	
**Liver enzymes**									
ALT	21.50 ± 10.27	50.33 ± 39.89	**0.023**	23.90 ± 8.79	47.06 ± 35.10	0.073	26.21 ± 15.26	45.07 ± 39.62	0.226
AST	24.51 ± 4.83	40.57 ± 35.67	0.146	24.10 ± 4.52	34.09 ± 16.78	0.105	23.57 ± 5.75	33.11 ± 22.02	0.267
GGT	25.12 ± 13.59	52.48 ± 32.31	**0.009**	21.49 ± 11.42	50.59 ± 30.88	**0.013**	30.00 ± 19.23	47.19 ± 28.50	0.138

Pre= baseline, Mid= after the control period/before XOS, Post= after XOS treatment WC= waist circumference, FFA= free fatty acids. Significant differences between the groups were analyzed with MANOVA and estimated marginal means and are highlighted in bold. Due to the group differences in body composition at baseline, the group comparisons at Mid and Post were conducted with ANCOVA using the corresponding baseline variable as covariate (adjustment is indicated with ^#^) and Bonferroni as *post hoc* test

### Differences in dietary intakes between the groups and changes during the study

Diet was recorded at baseline and post-treatment using validated 3-day food diaries [23]. Table 2 shows the dietary intakes in the IS and IR groups from those who returned the food diaries filled. The dietary intakes did not change during the study within-group nor were there differences in dietary intakes between the groups.

**Table 2:** Dietary intakes (Mean ± SD) at baseline (PRE) and after XOS treatment (POST)

Variable	PRE			POST		
	IS (*n* = 7) Mean ± SD	IR (*n* = 31) Mean ± SD	*p*	IS (*n* = 7) Mean ± SD	IR (*n* = 21) Mean ± SD	*p*
**Energy (kcal)**	2133 ± 531	2120 ± 439	0.957	1932 ± 460	1854 ± 366	0.708
**Fat** (g/day)	92.73 ± 30.09	102.27 ± 45.33	0.517	80.77 ± 31.91	88.04 ± 33.62	0.631
**SFA** (g/day)	35.19 ± 13.98	29.37 ± 8.24	0.375	33.09 ± 16.16	35.56 ± 15.87	0.743
**PUFA** (g/day)	14.31 ± 4.48	16.12 ± 5.15	0.384	11.78 ± 4.14	13.28 ± 7.69	0.526
**Carbs** (g/day)	188.79 ± 72.70	168.96 ± 73.23	0.546	184.90 ± 63.02	155.25 ± 100.26	0.382
**Protein** (g/day)	98.28 ± 22.68	90.05 ± 10.67	0.435	90.82 ± 17.55	92.24 ± 30.92	0.885
**Sucrose** (g/day)	34.70 ± 23.17	23.74 ± 8.68	0.265	30.56 ± 18.00	22.58 ± 18.52	0.350
**Fiber** (g/day)	24.30 ± 10.09	23.78 ± 8.99	0.908	21.14 ± 7.55	22.16 ± 8.09	0.778
**E% Fat**	39.60 ± 9.27	43.72 ± 16.63	0.755	37.69 ± 10.18	44.19 ± 19.02	0.270
**E% SFA**	15.00 ± 4.89	17.21 ± 11.21	0.430	15.34 ± 6.02	18.08 ± 9.44	0.396
**E% PUFA**	6.51 ± 2.44	7.18 ± 2.90	0.550	5.55 ± 1.50	6.68 ± 3.96	0.288
**E% Carbs**	39.39 ± 21.57	32.62 ± 12.23	0.464	38.55 ± 9.29	33.25 ± 20.18	0.359
**E% Protein**	18.96 ± 2.97	19.95 ± 3.98	0.502	19.51 ± 3.63	19.95 ± 3.98	0.800
**E% Saccharose**	6.59 ± 3.80	4.64 ± 1.65	0.228	6.28 ± 3.33	5.08 ± 4.56	0.479

SFA= saturated fats, PUFA= polyunsaturated fats, Carbs= carbohydrates, E%= energy percentage. Pre= baseline, Post= after XOS treatment. Significant differences between the groups were analyzed with MANOVA and estimated marginal means and are highlighted in bold

### Gut microbiota and their metabolites

**Fig. 2:**
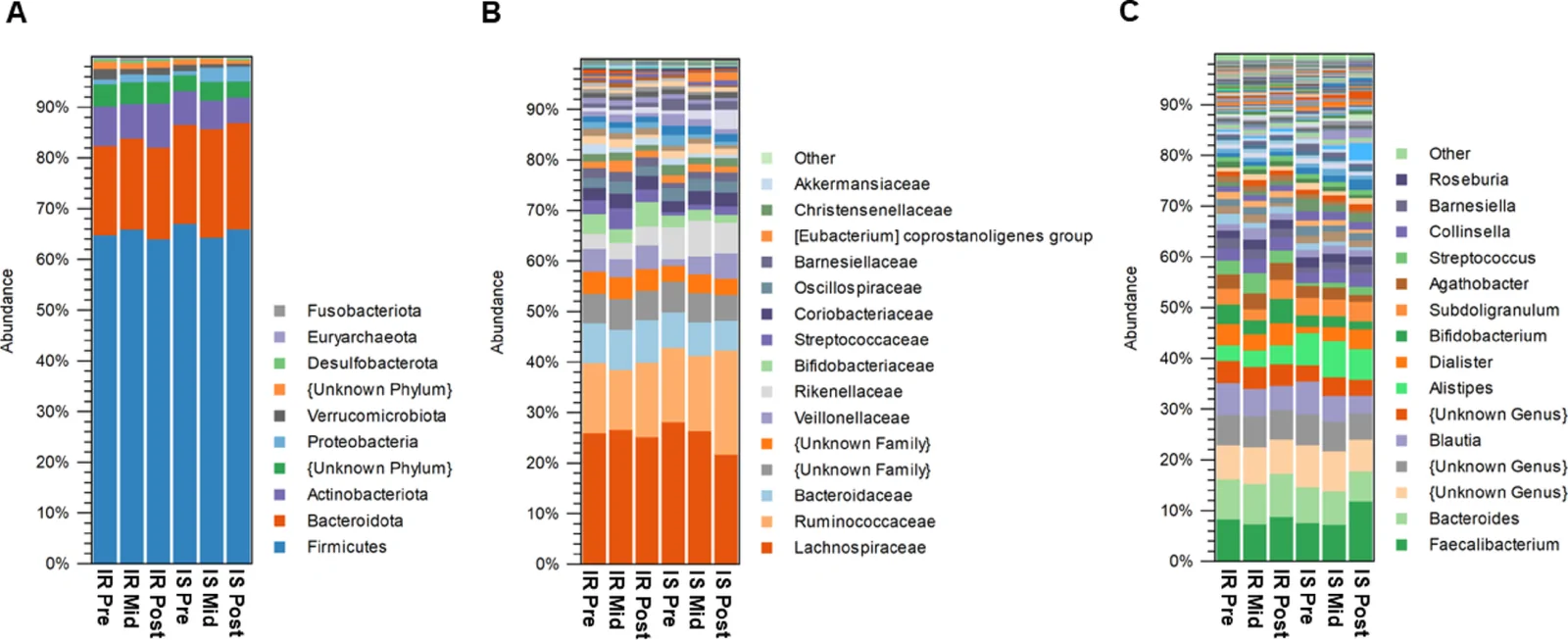
The overall gut microbiota composition at **A**) phylum, **B**) family and **C**) genus level. All detected phyla are shown, and of the families and genera the 15 more abundant. Pre= baseline, Mid= after the control period/before XOS, Post= after XOS treatment

**Fig. 3:**
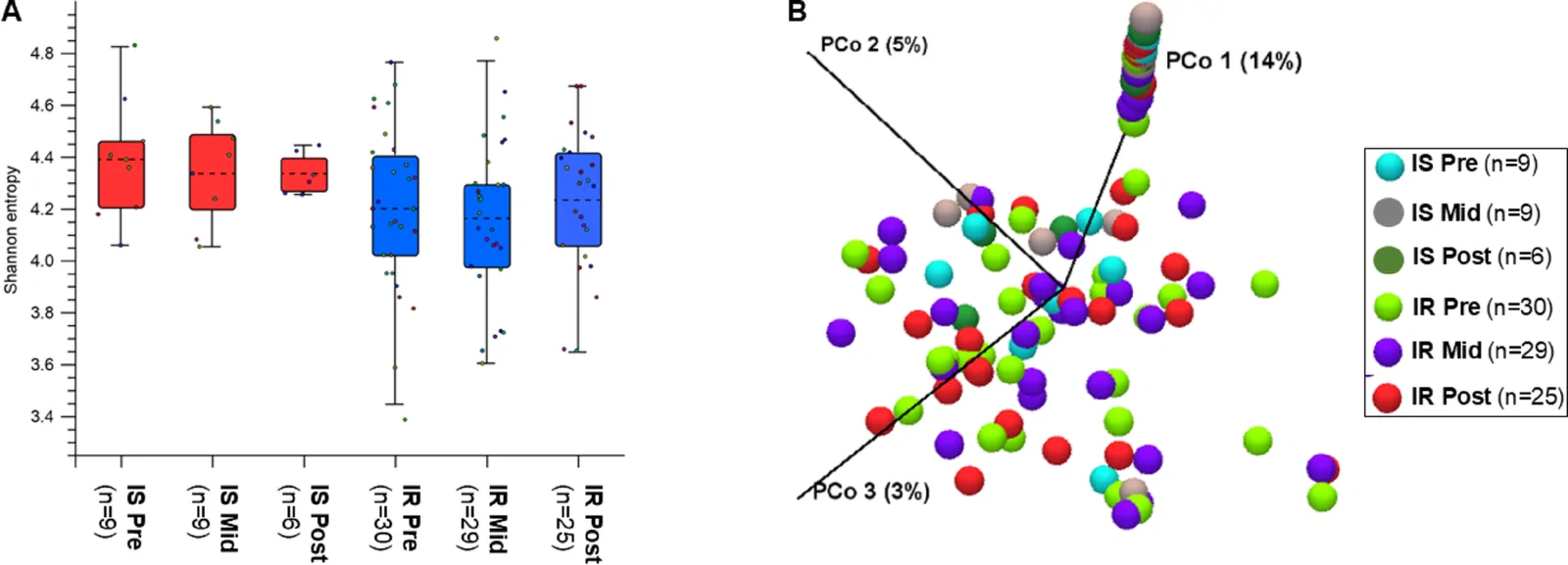
Gut microbiota diversity across the timepoints. **A**) Shannon index that indicates species diversity, and **B**) Principal component (PCo) plot of Bray Curtis distance. Pre= baseline, Mid= after the control period/before XOS, Post= after XOS treatment. The differences between the groups and timepoints in Shannon were analyzed with Kruskal-Wallis, and in beta-diversity with PERMANOVA

Figure 2A-C shows the average gut microbiota composition in the IS and IR groups at all timepoints. We found no changes or differences between the groups in gut microbiota alpha-diversity (Shannon index that indicates species diversity, Fig. 3A) or beta-diversity based on Bray Curtis distance and PERMANOVA for significance testing (Fig. 3B).

**Fig. 4:**
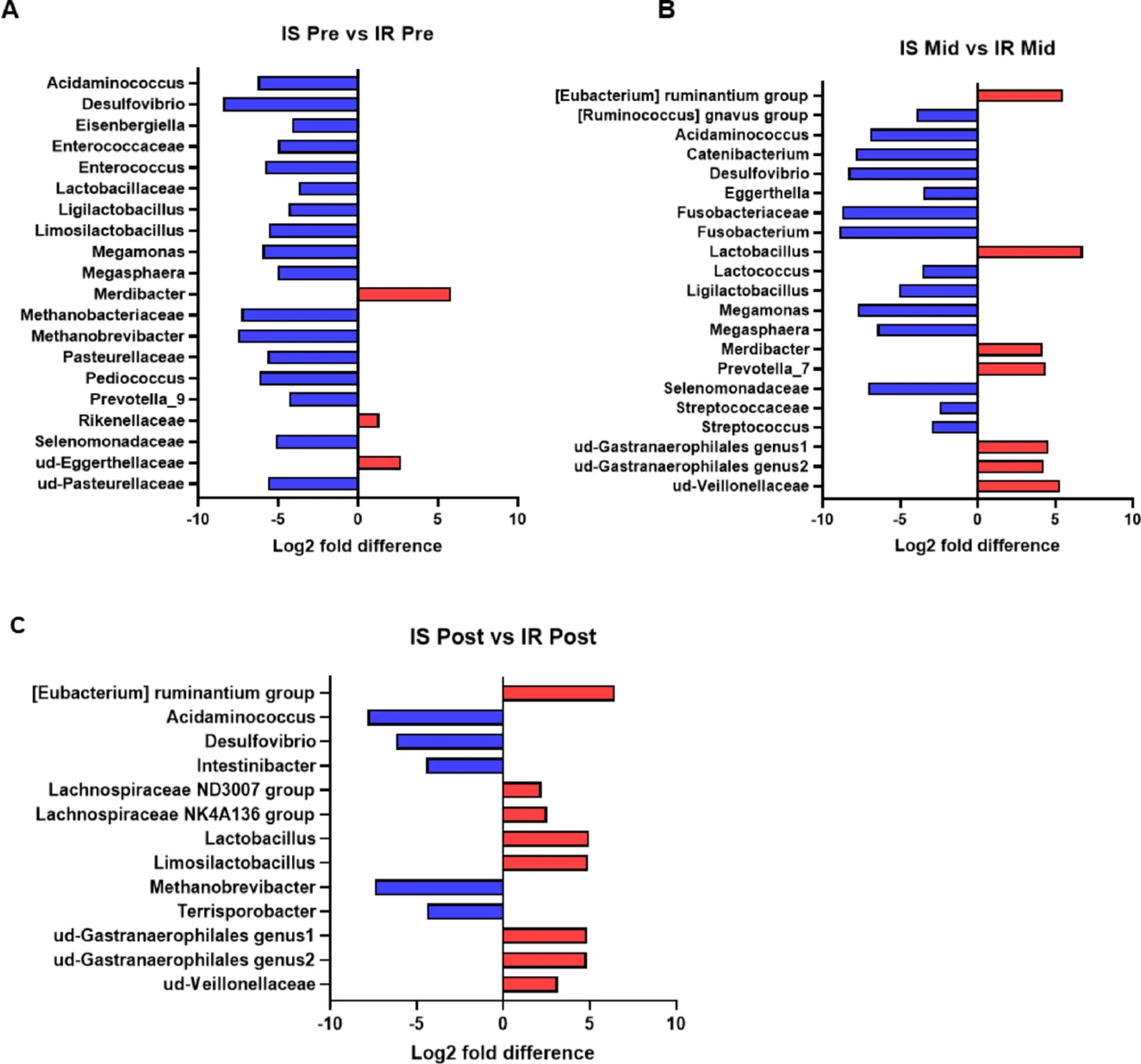
Differentially abundant taxa between the IS and IR groups at **A**) baseline (Pre), **B**) after the control period (Mid), and **C**) after XOS treatment (Post). ud= unidentified. Generalized Linear Model (GLM) followed by FDR correction was used for group comparisons

We then analyzed the differences in the microbial community composition between the groups at each timepoint. At baseline, there were 20 differentially abundant taxa based on Generalized Linear Model (GLM) comparison (FDR < 0.05 for all, Fig. 4A). Among others, the IR group was characterized by higher abundance of Lactobacillaceae family and further higher *Ligilactobacillus* and *Limosilactobacillus* genera (Fig. 4A). In addition, Methanobrevibacteriaceae family was higher in the IR group, which was attributed to higher *Methanobrevibacter* genus (Fig. 4A). Lower abundance of *Merdibacter*, Rikenelleceae and unidentified Pasteurellaceae was detected in the IR group (Fig. 4A). After the control period (Mid), *Ligilactobacillus* continued to be higher and *Merdibacter* lower in the IR group among 21 differentially abundant taxa (Fig. 4B). *Acidaminococcus* abundance was higher in the IR group both at baseline (Fig. 4A) and after the control period (Fig. 4B).

The differences between the groups diminished during XOS treatment suggesting that XOS changed the microbial composition towards same direction (Fig. 4C). However, *Acidaminococcus* and *Methanobrevibacter* continued to be higher in the IR group (Fig. 4C), while on a contrary to the baseline and after the control period, *Limosilactobacillus* and *Lactobacillus* were lower, respectively (Fig. 4C).

**Fig. 5:**
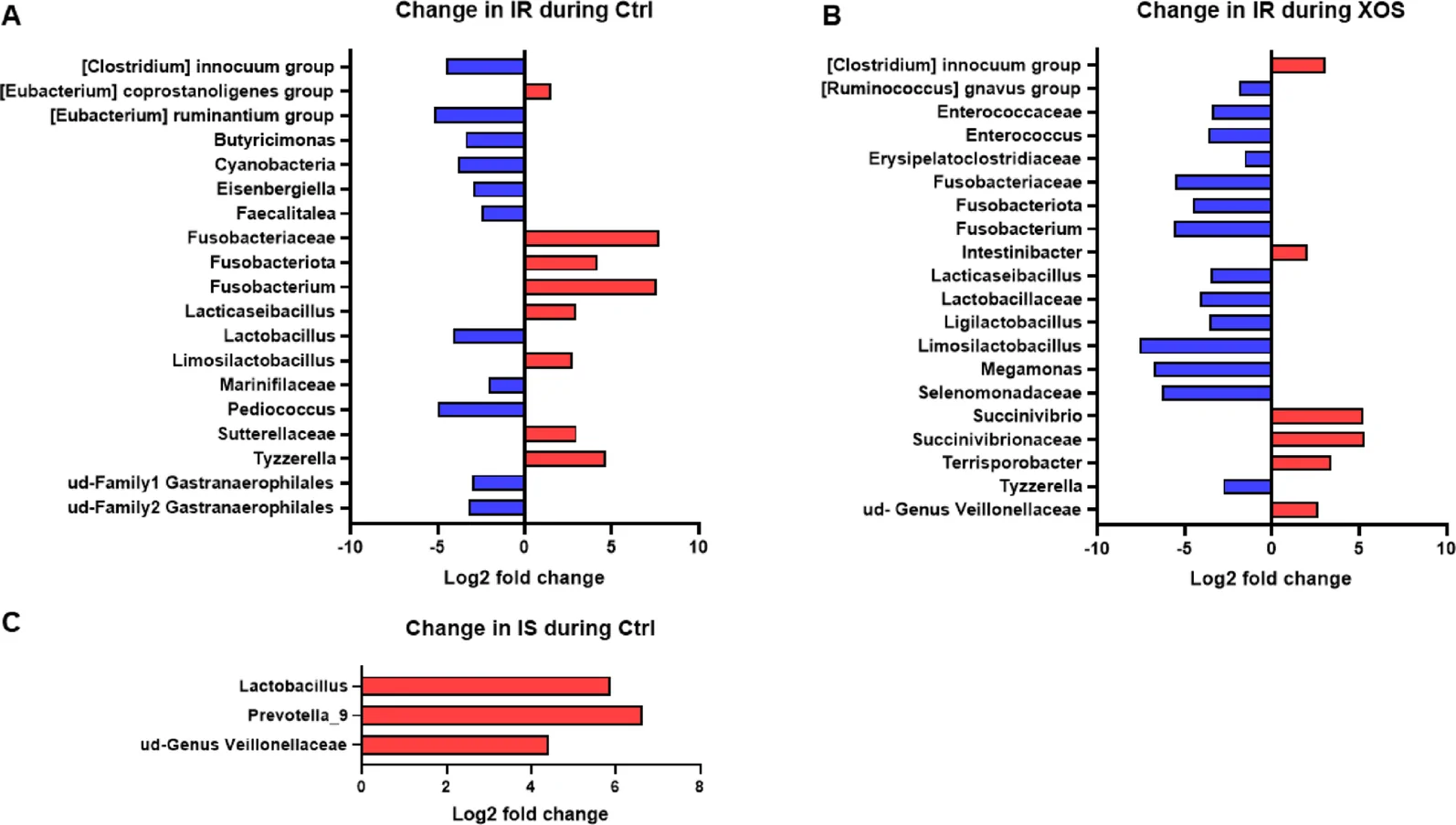
Longitudinal changes in the gut microbiota composition **A**) during the control period (Ctrl) in the IR group, **B**) during XOS treatment in the IR group, and **C**) during the control period (Ctrl) in the IS group. No taxonomic changes were detected during XOS treatment in the IS group. Generalized Linear Model (GLM) followed by FDR correction was used to analyze longitudinal changes. ud= unidentified

Next, we studied longitudinal changes in the gut microbiota community composition in both groups. We found that already during the control period the abundance of 19 taxa changed in the IR group and three in IS group (Fig. 5A&C).

Among these, Fusobacteriota and further Fusobacteriaceae and *Fusobacterium* increased in the IR group during the control period (Fig. 5A) but decreased during XOS treatment (Fig. 5B). In addition, the abundance of *Lacticaseibacillus* increased during the control period and decreased during XOS treatment in the IR group, while *Limosilactobacillus* increased during control period but decreased during XOS treatment (Fig. 5A & B). In the IS group, *Lactobacillus*, *Prevotella 9* and an unidentified genus of Veillonellaceae increased during the control period (Fig. 5C), while no changes were seen during XOS treatment. On a contrary, in the IR group *Lactobacillus* decreased during the control period (Fig. 5A) and an unidentified genus of Veillonellaceae family increased during XOS treatment but not control period (Fig. 5B).

**Fig. 6:**
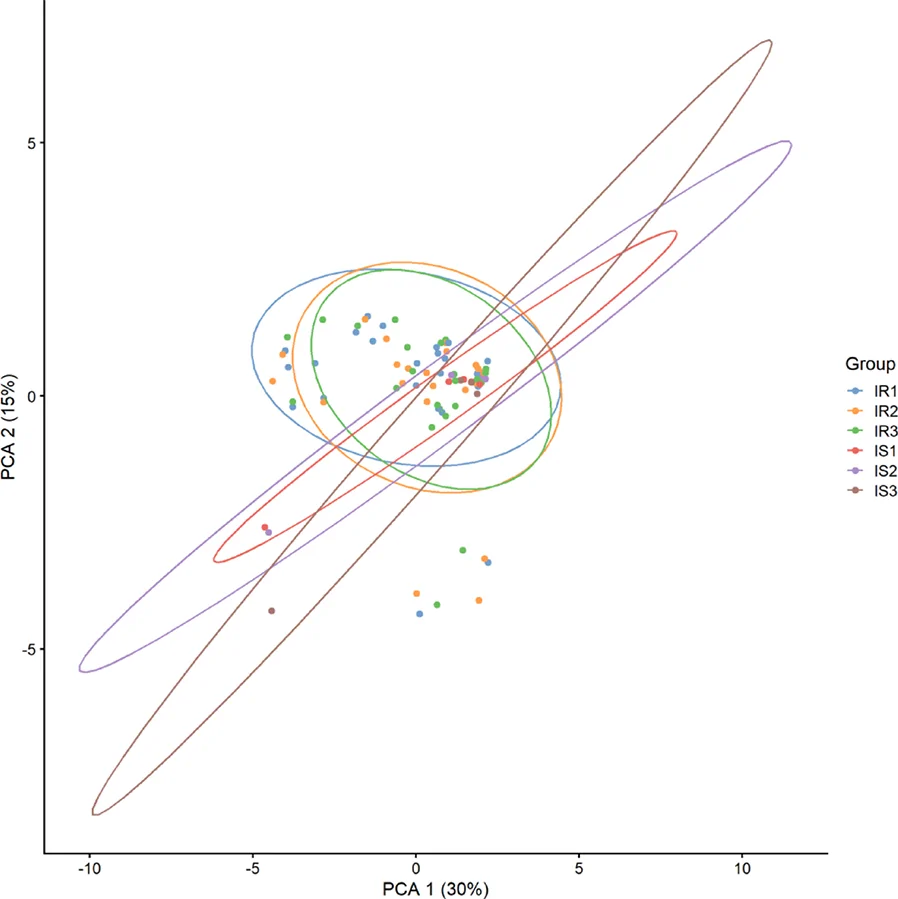
Principal component (PC) analysis of the 49 fecal metabolites across the timepoints. Log2-transform and z-scaling were applied to the data. 1 = baseline, 2 = after the control period, and 3 = after the XOS treatment

**Fig. 7:**
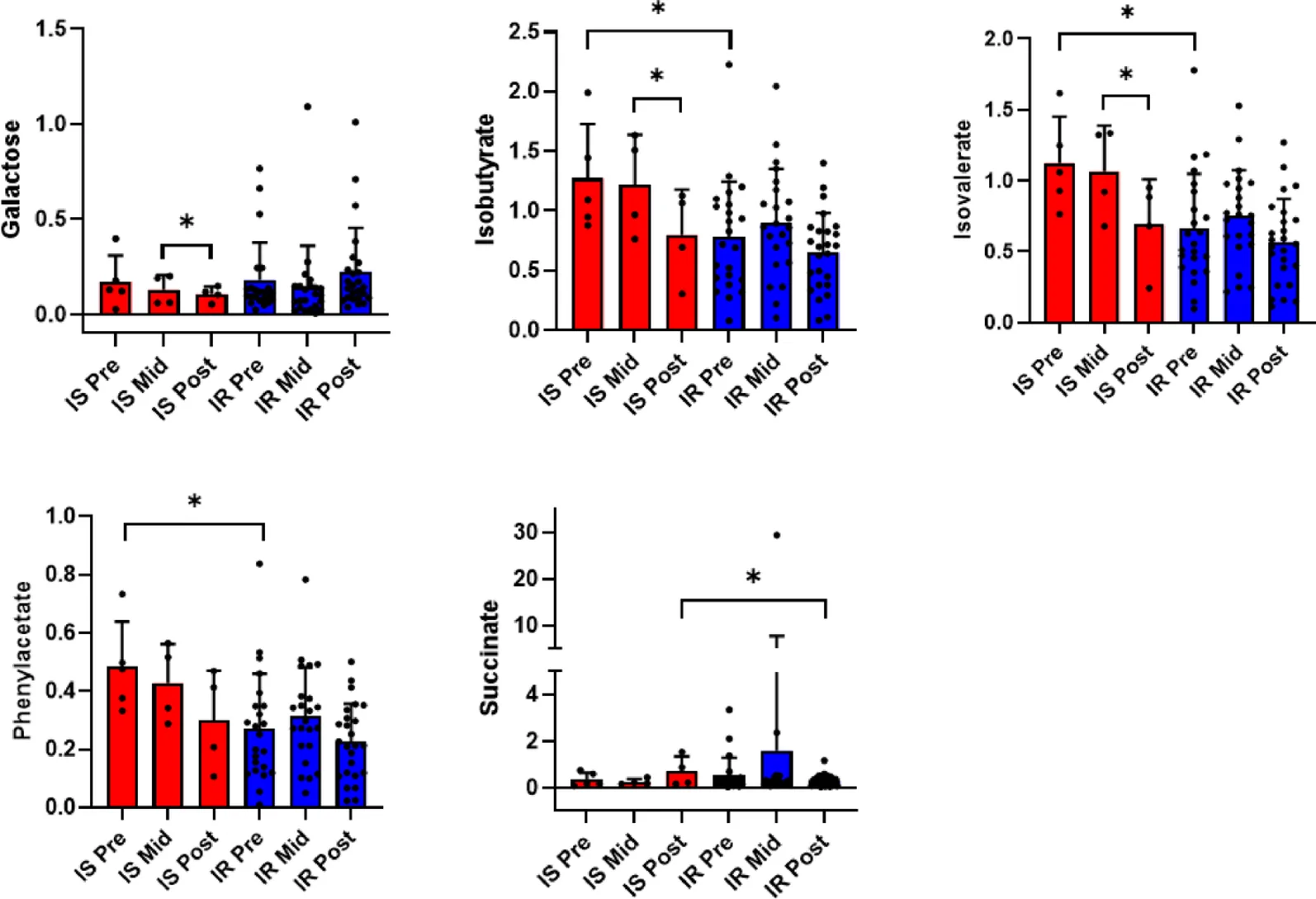
Significant differences in the fecal metabolites between the groups and within the groups across timepoints. Pre= baseline, Mid= after the control period/before XOS, Post= after XOS treatment. Group differences were analyzed with MANOVA with estimated marginal means and longitudinal changes with Wilcoxon signed-rank test. * *p* < 0.05

Fecal metabolites were quantified with ^1^H-NMR spectroscopy. A total of 49 metabolites were identified. First, to visualize group and timepoint differences in the metabolite dataset we used Principal Component Analysis (PCA). In Fig. 6, it can be observed that there are no clear overall differences. However, when analyzing individual metabolites, we observed that the concentration of galactose (*p* = 0.028), isobutyrate (*p* = 0.044) and isovalerate (*p* = 0.044) decreased during XOS treatment in the IS group while no changes were seen in the IR group (Fig. 7). Both isobutyrate (*p* = 0.042) and isovalerate (*p* = 0.020) levels as well as phenylacetate (*p* = 0.028) were lower in the IR group at baseline compared to IS (Fig. 7). In addition, the concentration of succinate was lower in the IR than IS group at the end of the XOS treatment (*p* = 0.006, Fig. 7).

### Cytokines

**Fig. 8:**
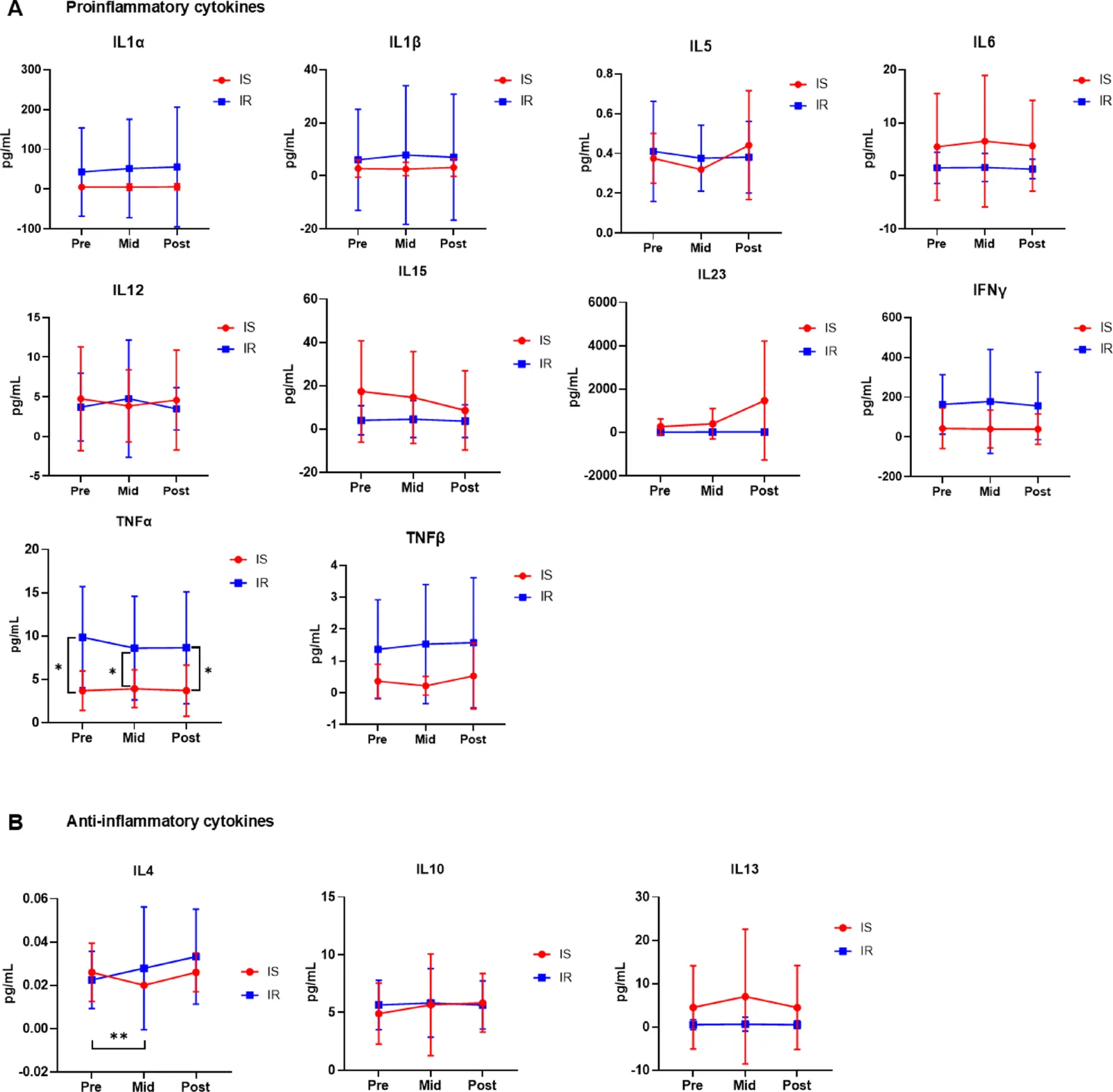
Serum levels of **A**) proinflammatory cytokines, and **B**) anti-inflammatory cytokines. Pre= baseline, Mid= after the control period/before XOS, Post= after XOS treatment. The dashed vertical line is intended to separate the groups from each other. Group differences were analyzed with MANOVA with estimated marginal means and longitudinal changes with Wilcoxon signed-rank test. *p < 0.05, **p < 0.01

15-plex ELISA and Quansys instrument were used to quantify serum levels of cytokines. We found that the level of pro-inflammatory cytokine TNFα was higher in the IR group at all timepoints (*p* < 0.05 for all, Fig. 8A). No longitudinal changes were found in pro-inflammatory cytokines. Of the anti-inflammatory cytokines, IL4 levels increased in the IR group during the control period (*p* = 0.004) with no change during XOS treatment similar to IL10 and IL13 (Fig. 8B).

## Discussion

In this within-subjects study design with a 1-month baseline control period, we studied insulin sensitive (IS) and resistant (IR) participants with overweight and obesity to see the effects of 4-months of prebiotic xylo-oligosaccharides (XOS) supplementation. We hypothesized that IR participants would benefit from XOS in terms of increasing insulin sensitivity as well as displaying a positive shift of gut microbiota composition accompanied by higher fecal SFCA metabolites and lower inflammatory response biomarkers. The main findings of this study are differences between IR and IS participants observed in gut microbiome, body composition, blood glucose and insulin profiles along with modest differences in proinflammatory cytokines. We report that four months of XOS supplementation did not affect insulin sensitivity or other variables substantially. However, we detected that XOS slightly decreased blood total cholesterol level and increased HDL in IR participants. Yet, HDL previously decreased in the IR group during the control period. In addition, fecal galactose, isobutyrate and isovalerate levels decreased in IS participants during XOS treatment, whereas microbial abundance changes were found only in IR participants with no changes in alpha- or beta-diversity. During the whole study period, body weight and BMI decreased in IR participants.

The main aim of our study was to increase the evidence on whether XOS supplementation enhances insulin sensitivity in humans with overweight. We found that XOS did not affect fasting glucose or insulin levels, and concomitantly no changes were seen in HOMA-IR or Matsuda index. Our findings agree with the study by Yang et al. in healthy and prediabetic adults using 2 g/day XOS [15] and the study by Chung et al. in healthy elderly using 4 g/day XOS [26] but disagree with the study by Huey-Herng Sheu et al. in T2D patients using 4 g/day XOS [14]. Thus, it remains still unclear whether XOS could improve insulin sensitivity in different populations, especially because the sample sizes of the above studies and ours were rather small and the studies used different doses of XOS.

In our study, the IR group showed higher values for weight, BMI, waist circumference, visceral fat area, total fat, and android mass at baseline compared to the IS group. This is well in line with a vast amount of literature (for review [27]. Nevertheless, when adjusting to the baseline values, the group differences in body composition disappeared after the control period, but again after XOS the IR group had higher weight, BMI, waist circumference, visceral fat area and total fat mass During the whole study, body weight decreased on average 1.35 kg and BMI 0.68 in the IR group but not in IS. However, this was not solely due to XOS treatment as the weight started to decrease already during the control period. In the studies of Yang et al. [15] and Huey-Herng Sheu et al. [14] XOS did not affect body weight or BMI. However, a synbiotic combination containing *Bifidobacterium animalis* subsp. *lactis*, galacto-oligosaccharides and XOS has been shown to reduce body fat percentage and waist circumference [28]. Another synbiotic, prebiotic XOS combined with probiotic MegaDuo^®^ *Bacillus subtilis* HU58 and *Bacillus coagulans* SC-208 reduced visceral adipose tissue by 35% [29]. It appears that XOS alone has not been widely studied in relation to body composition changes, while a meta-analysis shows that many other prebiotic oligosaccharide supplementations are accompanied by a significant decrease in weight, BMI, waist circumference, fasting blood insulin, and HOMA-IR compared to the control group [30]. Interestingly, we observed that XOS treatment slightly decreased total cholesterol levels, which however stayed above the normal range, and increased HDL to be within the normal range. At all timepoints, HDL levels were lower in the IR group. Similar observations on the effects of XOS on total cholesterol [14, 31] and HDL have been made also by other researchers [32]. Importantly, body composition, total cholesterol and HDL changes occurred without any changes in dietary intakes during our study. Serum GGT, a marker of liver damage that plays a role in the metabolism of glutathione and the transfer of amino acids across cell membranes decreased in the IR group during the study. Recently, GGT has transitioned from being solely a valid marker for alcohol consumption or chronic alcohol use to being recognized as part of a set of biomarkers indicating cardiometabolic risk, including IR [33].

Our study demonstrated 12 differently abundant bacterial genera between the groups at baseline, and 15 after the control period despite no differences in dietary intakes or gut microbiota diversity indices. Of these genera 5 were the same in both timepoints: *Desulfovibrio*, *Lactobacillu*s, *Megamonas*, *Megasphaera* and *Merdibacter*, all normal commensal gut microbes. After the XOS treatment 13 genera were differently abundant. However, many family levels differences disappeared suggesting that XOS changed the microbial composition towards more uniform “XOS intervention-specific” signature.

In this study, we reported a higher abundance of *Methanobrevibacter* in the IR group at baseline. *Methanobrevibacter* is an archaeal genus commonly found in the human gut and has been associated with IR, although its precise role remains under investigation. Some studies report a positive correlation between *Methanobrevibacter* and IR [34], while others suggest that this microbe contributes to inflammation [35]. Indeed, besides our study, a small pilot study compared individuals whose breath tests were positive for methane (reflecting colonization by *Methanobacter*) with those who tested negative [36]. Methane excretion correlated with impaired glucose tolerance independent of baseline insulin levels. In another small study, reducing *Methanobacter* levels with antibiotics associated with improvements in fasting glucose tolerance and related prediabetes indicators [37].

The abundance of *Fusobacterium*, an opportunistic pathogen, increased in the IR group during the control period and the abundance was higher compared to the IS group, but it decreased during XOS treatment. Our results are in line with a human intervention study showing that a reduction in *Fusobacterium* is associated with improved metabolic outcomes [38]. In addition, in observational cohorts, higher *Fusobacterium* presence appears linked to metabolic syndrome, potentially reflecting or contributing to IR [39]. While usually commensal, *Fusobacterium* can cause serious infections when it translocates to the circulation or host immunity is compromised [40]. In metabolic syndrome, *Megamonas* was enriched [39] as it was in our IR participants displaying characteristics of metabolic syndrome.

Somewhat surprisingly, some lactic acid bacteria were found to be more abundant in the IR group and their abundance decreased during XOS treatment. *Limosilactobacillus* abundance was higher in the IR group at baseline and after the XOS treatment and *Ligilactobacillus* at baseline and after the control period. *Limosilactobacillus* was created in 2020 by splitting from *Lactobacillus*. It has been shown to ameliorate IR and T2D [41]. Similarly, *Ligilactobacillus* (formerly *Lactobacillus*) strains, such as *L. acidophilus*, may help improve IR through various mechanisms, including reducing inflammation [42]. Thus, our results from comparing IR and IS groups seem contradictory considering the health benefits of these genera.

We hypothesized that fecal SCFAs resulting from nondigestible oligosaccharide fermentation by SCFA-producers could increase and associate with a decline of proinflammatory cytokines. Despite the increase in the abundance of SCFA-producers *Clostridium* innocuum group, *Intestinibacte*r, *Succinivibrio* and an unidentified genus of Veillonellaceae during XOS treatment, no changes in fecal SCFA levels were observed. Instead, we observed that fecal isobutyrate, isovalerate and phenylacetate were lower in the IR group at baseline compared to IS. In addition, the concentration of fecal succinate was lower in the IR than IS group at the end of the XOS treatment. In the IS group, fecal galactose, isobutyrate and isovalerate levels decreased during XOS treatment. Similarly, higher galactose has been reported to be associated with IR [43]. Contrarily, in a longitudinal cohort of 219 non-Hispanic White participants and 126 African American participants the lower levels of these metabolites have been associated with higher fasting glucose [44]. However, in accordance with our results showing lower levels of succinate in the IR group, GM-produced succinate has been shown to improve glucose homeostasis [45, 46].

Ultimately, we also analyzed the levels of different pro- and anti-inflammatory cytokines. The only differences found during the study were higher levels of TNF-α in the IR group at all timepoints and an increase in IL4 during the control period. In agreement, a previous study showed that plasma TNF-α correlates with IR in T2D patients, independent of obesity [47]. This may be due to that TNF-α triggers serine phosphorylation of insulin receptor substrate-1 (IRS-1), impeding its normal tyrosine phosphorylation. This disrupts insulin receptor signaling, forming a direct molecular link to IR [48].

This study is not without limitations. The sample size is rather small, especially in the IS group, that hinders the generalization of the results. However, age and sex were homogeneously distributed in the groups, which facilitates the reliability of the group comparisons. The strength is that diet was carefully monitored and there were no differences between the groups or changes within the group in the dietary intakes during the study. This is especially important when studying gut microbiota. It should be acknowledged that the group differences and changes in gut microbiota were only seen as modest taxonomic shifts with no significant changes in alpha- or beta-diversity. Therefore, the data does not support a global shift in microbial community structure in response to XOS. Physical activity was not recorded during the study and there is a possibility that the groups differed in their activity levels. Regarding cardiometabolic risk profiles, the IR group had higher abdominal obesity and lower HDL than IS at all timepoints. Thus, we cannot exclude that this may have affected some outcomes. In the future, more controlled and larger XOS interventions should be conducted.

## Conclusions

Key findings of this study are that IR and IS groups differed from each other in microbiome, body metrics, and glucose-insulin profiles, with minor cytokine differences. Interestingly, IS participants had decreased fecal galactose, isobutyrate, and isovalerate, while microbial taxonomic shifts occurred only in IR participants in response to XOS treatment. XOS modestly altered lipid parameters in the IR participants but did not significantly improve insulin sensitivity or inflammatory markers.

## Data Availability

The access to the data is restricted due to personal information protection (General Data Protection Regulation (GDPR) 2016/679 and Directive 95/46/EC). However, it is possible to contact author to ask for a copy of the material. The metadata of the larger study of which data was used for this work can be found in https://doi.org/10.17011/jyx/dataset/85068.

## Abbreviations

XOS: Xylo-oligosaccharides
T2D: type 2 diabetes
IR: insulin resistance
IS: insulin sensitivity
HOMA-IR: Homeostatic Model Assessment for Insulin Resistance
BMI: body mass index
NMR: nuclear magnetic resonance spectroscopy
SCFA: short-chain fatty acids
OGTT: Oral glucose tolerance test
IL: interleukin
IFN: interferon
TNF: tumor necrosis factor
OTU: operational taxonomic unit
PERMANOVA: Permutational Multivariate Analysis of Variance
